# Association between Preexisting Sleep Disorders and Oncologic Outcome in Patients with Oral Cavity Squamous Cell Carcinoma: A Nationwide Propensity Score—Matched Population-Based Cohort Study

**DOI:** 10.3390/cancers14143420

**Published:** 2022-07-14

**Authors:** Shih-Hao Ou, Wan-Ming Chen, Ben-Chang Shia, Szu-Yuan Wu, Hsuan-Chih Lin

**Affiliations:** 1 Department of Otorhinolaryngology, Lo-Hsu Medical Foundation, Lotung Poh-Ai Hospital, Yilan 265, Taiwan; asklepios790423@gmail.com; 2 Graduate Institute of Business Administration, College of Management, Fu Jen Catholic University, Taipei 242, Taiwan; daisywanmingchen@gmail.com (W.-M.C.); 025674@mail.fju.edu.tw (B.-C.S.); 3 Department of Food Nutrition and Health Biotechnology, College of Medical and Health Science, Asia University, Taichung 413, Taiwan; 4 Big Data Center, Lo-Hsu Medical Foundation, Lotung Poh-Ai Hospital, Yilan 265, Taiwan; 5 Division of Radiation Oncology, Lo-Hsu Medical Foundation, Lotung Poh-Ai Hospital, Yilan 265, Taiwan; 6 Department of Healthcare Administration, College of Medical and Health Science, Asia University, Taichung 413, Taiwan; 7 Cancer Center, Lo-Hsu Medical Foundation, Lotung Poh-Ai Hospital, Yilan 265, Taiwan; 8 Centers for Regional Anesthesia and Pain Medicine, Taipei Municipal Wan Fang Hospital, Taipei Medical University, Taipei 110, Taiwan

**Keywords:** distant metastasis, locoregional recurrence, OSCC, sleep disorder, survival

## Abstract

**Simple Summary:**

We aimed to estimate the effects of preexisting sleep disorders on the oncologic outcomes of patients with oral squamous cell carcinoma (OSCC) after receiving standard treatments. We conducted a head-to-head propensity-score-matching-based study to mimic a randomized trial to compare the survival, locoregional recurrence, and distant metastasis rates between OSCC patients with and without sleep disorders. The patients with sleep disorders receiving curative treatments for OSCC had poorer oncologic outcomes than did those without sleep disorders. Preexisting sleep disorders may be survival predictors in patients with OSCC. Studies on how pharmacological and behavioral treatments for sleep problems improve survival benefits in patients with OSCC are warranted.

**Abstract:**

Purpose: To investigate the effects of preexisting sleep disorders on the oncologic outcomes of patients receiving standard treatments for oral squamous cell carcinoma (OSCC). Methods: The patients recruited from the Taiwan Cancer Registry Database who had received surgery for stage I–IVB OSCC. The Cox proportional hazards model was used to analyze all-cause mortality, locoregional recurrence (LRR), and distant metastasis (DM). The patients were categorized into those with and without sleep disorders (Groups 1 and 2, respectively) through propensity score matching. Results: In the multivariate Cox regression analysis, the adjusted hazard ratios for all-cause mortality, LRR, and DM for Group 1 compared with Group 2 were 1.19 (95% confidence interval (CI): 1.04–1.36; *p* = 0.011), 1.47 (95% CI: 1.23–1.75; *p* < 0.001), and 1.15 (95% CI: 1.02–1.44; *p* = 0.025), respectively. Conclusion: OSCC patients with sleep disorders demonstrated poorer oncologic outcomes than did those without sleep disorders. Therefore, before OSCC surgery, patients with OSCC should be screened for preexisting sleep disorders because they may serve as predictors for survival in these patients. Future studies investigating the survival benefits of pharmacological and behavioral treatments for sleep problems in patients with OSCC are warranted.

## 1. Introduction

Sleep disorders are prevalent in patients with head and neck cancer (HNC) before, during, and after HNC treatment [1,2]. The prevalence of sleep disorders before and after HNC treatment has been reported to be 51–66% [1]. According to a recent systemic review and meta-analysis, patients with oral squamous cell carcinoma (OSCC) have sleep problems before receiving OSCC treatment [1]. Moreover, OSCC treatment may aggravate these sleep disturbances; with prolongation of treatment time, these symptoms may exacerbate, and they may persist even after treatment cessation [3].

The association of sleep with all-cause mortality risk in the general population has received substantial research attention [4,5,6,7]. However, despite more than half of cancer survivors having sleep problems—a much higher prevalence than that reported in the general population—knowledge regarding the sleep–cancer survival association is scant [2,8,9,10]. Although patients with HNC with regular prediagnostic sleep problems may have a higher mortality risk, the data on the sleep disorder–HNC survival relationship remains unknown [11]. Thus far, no study has investigated the influence of sleep quality on mortality, locoregional recurrence (LRR), and distant metastasis (DM) risks in patients with HNC. The potential mechanisms underlying the sleep-disorder-accelerated HNC progression to recurrence and metastasis, which contribute to mortality, remain unclear. One of these mechanisms is immune system dysregulation; experimental sleep deprivation has been noted to significantly reduce natural killer cell activity in healthy humans [12], and inflammation has been linked to cancer prognosis [13]. A study examining the sleep–oncologic outcome association in patients with HNC is required; its findings may aid in improving the patients’ quality of life and long-term survival rates.

To control for factors such as HNC treatment, prognosis, and survival in patients, here, we focused only on patients with oral cavity squamous cell carcinoma (OSCC) [14,15,16,17,18]. Specifically, we assessed whether a preexisting sleep disorder is an independent risk factor for mortality, LRR, and DM in OSCC patients who underwent curative surgery followed by adjuvant treatments stipulated in the National Comprehensive Cancer Network (NCCN) guidelines [19]. By identifying the association between preexisting sleep disorders and survival in patients with OSCC, we may be able to understand sleep disorders in patients with OSCC further and provide evidence-based knowledge for future research to eventually improve the survival of OSCC patients with sleep disorders; in particular, health-care providers may benefit from our findings.

## 2. Patients and Methods

### 2.1. Study Population

Patient data analyzed in this study were retrieved from the Taiwan Cancer Registry Database (TCRD), a nationwide registry containing detailed information such as each patient’s cancer status and stage, cigarette-smoking and betel-nut-chewing habits, treatment modalities, pathologic data, irradiation doses, and chemotherapy regimens used [16,17,20,21]. There were 8166 enrolled patients if they received a diagnosis of American Joint Committee on Cancer (AJCC) clinical stage I–IVA OSCC between 1 January 2009 and 31 December 2018 before the inclusion and exclusion criteria. The index date was set as the date of curative surgery for OSCC. The follow-up duration was measured from the index date to 31 December 2019. The study protocols were reviewed and approved by the Institutional Review Board of the Tzu-Chi Medical Foundation (IRB109-015-B).

### 2.2. Inclusion and Exclusion Criteria

The pathological information of all patients enrolled in this study was reviewed. Only patients with newly diagnosed OSCC but no other cancers or distant metastases; those aged ≥ 20 years; and those who received curative surgery at the OSCC pathologic stage I–IV, defined according to the AJCC Cancer Staging Manual, 7th Edition, were included. All the included patients received curative-intent surgery including tumor resection, neck lymph node dissection, or both. Adjuvant treatments such as adjuvant concurrent chemoradiotherapy (CCRT) and adjuvant radiotherapy (RT) were guided and performed with adherence to the NCCN guidelines [19].

We excluded patients with a history of cancer before the receipt of OSCC diagnosis, unclear pathologic staging, histological types other than squamous cell carcinoma, or an unclear Charlson comorbidity index (CCI), as well as those without known cigarette-smoking or betel-nut-chewing habits or sex data. Patients were also excluded if they had undergone nonstandard adjuvant RT (i.e., irradiation to tumor bed and high risky regional lymph nodes at doses <60 Gy). The chemotherapy regimens administered concurrently with RT in our study were platinum-based regimens with a total cumulative dose of ≥240 mg/m^2^ at least [21]. The CCI was implemented to measure comorbidity incidence. These comorbidities were coded on the basis of the International Classification of Diseases, Ninth Revision, Clinical Modification (ICD-9-CM). The CCI included comorbidities reported and assigned at the first admission or after more than two outpatient department visits. Comorbidities were counted only if they were observed within 12 months before the index date.

The enrolled patients were categorized as having sleep disorders 1 year before the index date (to remove OSCC-related sleep disturbance) if an *ICD* (including *ICD-9-CM*) code or *ICD-10-CM* code corresponding to the third edition of the International Classification of Sleep Disorders (ICSD-3) had been assigned in two or more family medicine, neurology, or psychology outpatient visits or in at least one instance of hospitalization [22,23]. Patients were also deemed to have a sleep disorder if they had an *ICD* code corresponding to the *ICSD-3* and had been prescribed benzodiazepines for at least 30 days in the year before the index date. The *ICSD-3*, which includes 60 specific diagnoses within seven categories, is the primary reference used to sleep disorder diagnosis and classification [24,25]. Sleep disorders included in the *ICSD-3* can be categorized into seven primary categories: insomnia disorders, sleep-related breathing disorders, central disorders of hypersomnolence, circadian rhythm sleep–wake disorders, sleep-related movement disorders, parasomnias, and other sleep disorders. However, the enrolled patients’ sleep disorders were not subcategorized.

### 2.3. Propensity Score Matching and Covariates

Propensity score matching (PSM) was implemented to reduce confounding effects before comparing all-cause death between Groups 1 and 2. With a caliper width of 0.2, the two groups were matched at a 2:1 ratio for the following variables: age, sex, cancer locations (buccal, gingival, hard palate, oral tongue, and lip), pathologic AJCC stages, income levels, urbanization, CCI scores, alcohol liver diseases, cigarette smoking, betel nut chewing, adjuvant RT, and adjuvant CCRT [26]. We used a Cox regression model to regress all-cause mortality on various sleep disorder statuses with a robust sandwich estimator to account for clustering within matched sets [27]. Before statistical analysis, the potential predictors were controlled through PSM (Table 1). To determine potential independent predictors for all-cause death, sleep-disorder-status-associated hazard ratios (HRs), age, sex, cancer locations (i.e., buccal, gingival, hard palate, oral tongue, and lip), pathologic AJCC stages, income levels, urbanization, CCI scores, alcohol liver diseases, cigarette smoking, betel nut chewing, adjuvant RT, and adjuvant CCRT were calculated through multivariate time-dependent Cox regression analysis. The primary endpoint for Groups 1 and 2 was all-cause mortality, whereas LRR and DM were the secondary endpoints.

After we applied the inclusion and exclusion criteria and conducted PSM, we identified 2458 patients with OSCC who had undergone curative surgery for AJCC clinical stage I–IV OSCC and were eligible for further analysis. These patients were divided into two groups on the basis of their sleep disorder status to compare all-cause mortality, LRR, and DM. Patients with a sleep disorder diagnosed before curative surgery were placed into Group 1, whereas those without a diagnosis of a preexisting sleep disorder were placed into Group 2.

### 2.4. Statistical Analysis

We compared Groups 1 and 2 by applying the independent *t*-test for continuous variables and the chi-squared test for categorical variables. Continuous variables are summarized and described as means ± standard deviations. We compared differences in follow-up durations between the two groups by using the Mann–Whitney *U* test. The *p*-values for adjuvant RT and CCRT were produced using Gray’s test (Table 1). Comparative analysis was performed after the confounders were adjusted for. A *p*-value of <0.05 was considered to indicated statistical significance through a two-tailed Wald test. Survival curves were estimated using the Kaplan–Meier estimator. A comparison of survival, cumulative LRR, or DM curves—stratified according to matched sets—was performed to determine the differences between the two groups by using a stratified logrank test [28].

All statistical analyses were performed using SAS (version 9.4; SAS Institute, Cary, NC, USA).

## 3. Results

### 3.1. PSM and Study Cohort

After the inclusion and exclusion criteria were applied and PSM was conducted, 2458 patients with stage I–IVB OSCC (*n* = 828 and 1630 in Groups 1 and 2, respectively) were eligible for further analysis. The clinicodemographic characteristics of each group are summarized in Table 1. Because of PSM, the aforementioned covariates in Table 1 were well matched and demonstrated no statistically significant differences. The outcomes mortality, LRR, DM, follow-up duration, and sleep disorder status within 1 year before the index date were inconsistent between the two groups. The crude incidences of all-cause death in Groups 1 and 2 were 44.42% and 51.93%, respectively (*p* < 0.001).

### 3.2. All-Cause Death

The multivariate Cox regression for survival indicated the association of sleep disorder, older age (>50 years), male sex, high CCI score, low income level, advanced AJCC stage, cigarette-smoking habit, betel-nut-chewing habit, and alcohol liver diseases with poor overall survival (OS; Table 2). The adjusted HR (aHR; 95% confidence interval (CI)) for all-cause death in patients with sleep disorders compared with those without sleep disorders was 1.19 (1.04–1.36; *p* < 0.001). The aHRs (95% CIs) for all-cause death were 1.22 (1.03–1.45) and 1.67 (1.42–1.98) in patients aged 51–60 and >60 years, respectively, compared with those aged ≤50 years. The aHR (95% CI) for mortality was 1.19 (1.05–1.36) in patients with CCI ≥ 1 compared with those with CCI = 0 and 1.76 (95% CI: 1.50–2.06) for male patients compared with female patients. The aHRs (95% CIs) for mortality were 0.52 (0.33–0.83), 0.42 (0.25–0.70), and 0.28 (0.16–0.51) in patients with an income New Taiwan Dollar (NT$) 20,001–NT$30,000, NT$30,001–NT$45,000, and >NT$45,000 compared with those with a low income level, respectively. The aHRs (95% CIs) for mortality were 1.37 (1.03–1.82), 2.44 (2.02–2.95), and 2.86 (2.36–3.46) in patients with stage II, III, and IV OSCC compared with those with stage I OSCC, respectively. The aHRs (95% CIs) for mortality were 1.35 (1.16–1.58), 1.07 (1.02–1.14), and 1.27 (1.09–1.48) in patients who smoked, chewed betel nut, and had alcohol liver diseases compared with those who did not, respectively.

### 3.3. LRR

The multivariate Cox regression for LRR indicated that sleep disorder, male sex, advanced AJCC stage, cigarette-smoking habit, and betel nut chewing were associated with a high LRR risk (Table 3). In addition, adjuvant RT or adjuvant CCRT was associated with LRR risk reduction. The aHR (95% CI) for LRR was 1.47 (1.23–1.75) in patients with sleep disorders compared with those without sleep disorders. The aHRs (95% CIs) for LRR in patients who were male, had stage II OSCC, had stage III OSCC, had stage IV OSCC, smoked cigarettes, and chewed betel nut were 1.84 (1.43–2.37), 1.35 (1.08–1.7), 1.64 (1.25–2.15), 1.93 (1.60–2.33), 1.25 (1.02–1.54), and 1.26 (1.08–1.48), respectively. The aHRs (95% CIs) for LRR in patients receiving adjuvant RT and adjuvant CCRT were 0.69 (0.49–0.79) and 0.56 (0.41–0.83), respectively.

### 3.4. DM

The multivariate Cox regression for DM indicated that sleep disorder, male sex, and advanced AJCC stage were associated with DM risk (Table 4). The aHRs (95% CIs) for DM were 1.15 (1.02–1.44) in patients with sleep disorders compared with those without sleep disorders and 1.63 (1.22–2.18) in male patients compared with female patients. Moreover, the aHRs (95% CIs) for DM in patients with stage II, III, and IV OSCC compared with those with stage I OSCC were 1.89 (1.14–3.80), 3.31 (1.60–4.62), and 8.44 (6.58–9.55), respectively.

### 3.5. Kaplan–Meier Survival and Cumulative Incidence of LRR and DM in Patients with OSCC with and without Sleep Disorders before Surgery

**Figure 1:**
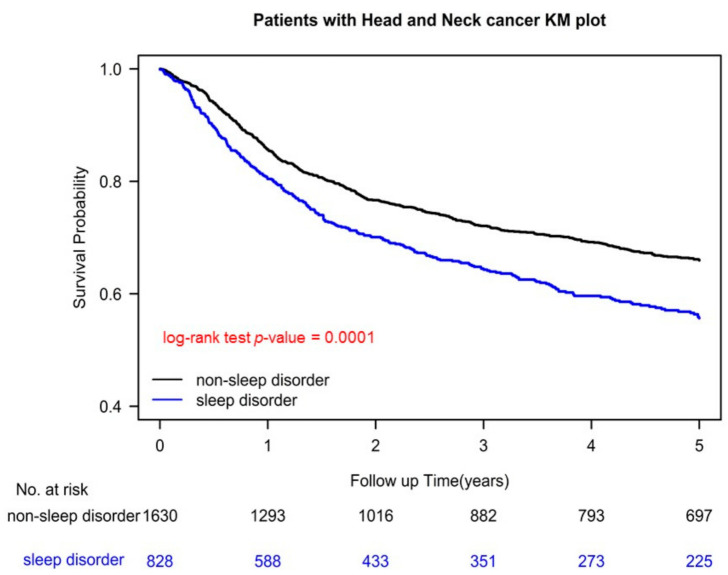
Kaplan–Meier survival curves of propensity-score-matched patients with oral cavity squamous cell carcinoma with and without sleep disorders.

According to our Kaplan–Meier curves, OSCC patients with sleep disorders diagnosed before surgery had shorter survival and significantly higher LR and DM cumulative incidence than did those without sleep disorders (all *p* < 0.001; Figure 1, Figures S1 and S2).

## 4. Discussion

This study revealed a significant relationship between preexisting sleep disorders and OSCC mortality, LRR, and DM risks (i.e., poor oncologic outcomes). Compared with patients without sleep disorders, those with a sleep disorder within 1 year before OSCC surgery had increased all-cause death, LRR, and DM risks. To the best of our knowledge, no previous head-to-head PSM study has estimated the OS, LRR, and DM risk in OSCC patients with preexisting sleep disorders after they received surgery and standard treatments according to the NCCN guidelines. This is the first head-to-head PSM that imitates a randomized controlled trial (RCT) design to balance all the confounding factors listed in Table 1 [29].

Performing an RCT to evaluate oncological outcomes in OSCC patients with and without preexisting sleep disorders undergoing curative surgery is difficult because sleep disorders cannot be treated using tangible interventions [30]. Traditionally, striking a balance among the confounding factors for death in OSCC patients with and without a sleep disorder (i.e., the case and control groups, respectively)—a main requirement of the RCT design—is not possible [30]. Therefore, a PSM-based design, such as that in the current study, can resolve this issue by maintaining balance among the confounding factors for the case and control groups—all without any bias. Moreover, PSM is currently the recommended standard tool for estimating the effects of covariates in studies where a potential bias may exist. Although the main advantage of the PSM methodology is the more precise estimation of the covariate effect, PSM cannot control for factors not accounted for in the model. Moreover, PSM is based on an explicit selection bias of those who could be matched; in other words, individuals who could not be matched are not part of the scope of inference.

As we noted in this study, a sleep disorder diagnosed 1 year prior to OSCC surgery is an independent risk factor for OS in patients diagnosed as having stage I–IV OSCC and receiving standard treatments (Table 2, Figure 1). Rather than focusing on preexisting or pretreatment sleep disorders, almost all relevant studies thus far have assessed all-cause death risk posed by sleep problems in patients after receiving an OSCC diagnosis. However, given that most sleep disorders begin before HNC diagnosis and precancerous sleep problems tend to linger and become aggravated during cancer treatment [1,8,31,32,33], our findings may be deemed consistent with those of studies reporting sleep problems to be a mortality risk factor in patients with OSCC [1,8,32,33,34]. In addition to these major findings, our study corroborates the findings of studies that have identified sleep disorder, older age (>50 years), male sex, high CCI, low income, advanced AJCC stage, cigarette smoking, betel nut chewing, and alcohol liver diseases as independent predictors for OS (Table 2) [16,17,20,21]. Moreover, our study is the first to demonstrate that preexisting sleep disorders are associated with increased LRR and DM risk in OSCC patients who receive standard treatments. Although the significance of the results presented in Table 2, Table 3 and Table 4 should be considered in future clinical practice and trials concerning OSCC and sleep disorders, further studies may be required to establish the mechanism underlying our findings.

Several factors may influence the cause of or mechanism underlying the association of sleep disorders with increased in all-cause death, LRR, and DM in OSCC patients receiving standard treatments. Sleep problems have been linked to increased mortality risk in the general population. Limited evidence suggests similar relationships among people diagnosed as having cancer, particularly in OSCC and sleep disorders before OSCC diagnosis [35]. Although immune system dysregulation may explain the association between sleep disorders and mortality, whether similar biological mechanisms explain the association of preexisting sleep disorders with all-cause death, LRR, and DM remain unclear. OSCC patients with sleep disorders have shorter survival, possibly because sleep disorders are associated with systemic inflammation [36,37], which is in turn associated with the pathways similar to those of OSCC progression [38,39]. Systemic inflammation markers, such as C-reactive protein, interleukin 6, and fibrinogen, have been linked to poor sleep [37]. Systemic inflammation is an independent predictor for clinical outcomes in patients with OSCC [40]. Furthermore, studies have shown that sleep disorder is associated with larger, more aggressive tumors in animal models, but not in humans [41]. Our study is the first clinical study demonstrating the association of preexisting sleep disorders with poor oncologic outcomes, including high mortality, LRR, and DM risk, in patients receiving standard OSCC treatments (Table 2, Table 3 and Table 4, Figure 1, Figures S1 and S2).

A major finding is that sleep disorder diagnosis 1 year before OSCC surgery is an independent prognostic factor for the survival of patients with OSCC after surgery because most OSCC patients with sleep disorders in our study received a sleep disorder diagnosis before receiving an OSCC diagnosis [1,33]. Here, patients without a diagnosis of a sleep disorder before OSCC surgery exhibited superior OS, LRR, and DM risks (Table 2, Table 3 and Table 4, Figure 1, Figures S1 and S2); therefore, routine screening for sleep disorders and offering appropriate treatment for patients with OSCC risk may be beneficial. According to an epidemiological study in Taiwan, the incidence of oral cancer was 123-fold higher in patients who smoked, consumed alcohol, and chewed betel quid than in those who did not [42]. Patients with preexisting sleep disorders and risk factors for OSCC form the population susceptible to having poor oncologic outcomes. Interventions facilitating sleep disorder discovery and treatment may improve survival after OSCC diagnosis in this population.

Our study has several strengths. It is the first, largest cohort study to estimate the effects of preexisting sleep disorders on the survival outcomes of OSCC patients undergoing standard OSCC treatments in accordance with the NCCN guidelines. In addition, from an oncology perspective, this study highlighted the necessity of sleep quality, for which a prospective RCT can likely not be conducted without ethical concerns. We also analyzed most prognostic factors identified in the literature—namely, mortality, LRR, and DM—in our patients with OSCC if the relevant data were available in the TCRD. Because PSM minimizes the confounding effect, the covariates between the groups with and without sleep disorders in our study were homogenous—all were stage I–IV OSCC patients receiving OSCC surgery and other standard treatments; no selection bias was noted between the two groups (Table 1).

The results of this study should be interpreted with caution because of the following limitations. First, as in all retrospective studies, residual or unmeasured confounding and selection bias were inevitable. Second, information that may affect the oncologic outcomes of patients with OSCC, such as dietary habits, socioeconomic status, or body mass index, are not collected in the TCRD. Third, our results and conclusions may be difficult to extrapolate to non-Asian populations because we enrolled only Asian patients in this study. Therefore, additional large-scale studies on the causal relationship between sleep disorders and OSCC survival rates in various ethnic groups are required. Fourth, the diagnoses of all comorbid conditions were based on *ICD-9-CM* or *ICD-10-CM* codes in this study. Nevertheless, because the Taiwan Cancer Registry Administration periodically audits files and penalizes hospitals with the evidence of abnormal charges or improper practices identified through medical chart reviews and patient interviews, the Taiwan Cancer Registry long-form data most likely have a high degree of accuracy [43]. Therefore, given the magnitude and statistical significance of the present results, our conclusions are unlikely to be affected by the aforementioned limitations.

## 5. Conclusions

In this study, OS, LRR, and DM were worse in OSCC patients with preexisting sleep disorders than in those without. Therefore, screening for and evaluation of preexisting sleep disorders before OSCC surgery are essential because these disorders may be presurgical predictors for oncologic outcomes in patients with OSCC. Additional studies on survival benefits of pharmacological and behavioral treatments for sleep problems in patients with OSCC are warranted.

## Figures and Tables

**Table 1:** Characteristics of propensity score-matched patients with and without sleep disorders before oral cavity squamous cell carcinoma diagnosis.

	Patients without Sleep Disorder		Patients with Sleep Disorder		*p*-Value	SMD
	N = 1630		N = 828			
	N	%	N	%		
Age (mean ± SD)	56.39 ± 12.88		57.42 ± 12.94		0.061	0.080
Age, median (IQR), years	55.84 (47.55–64.85)		56.16 (48.29–66.43)		0.188	
Age group, years	1630		828		0.965	0.011
≤50	509	31.23%	256	30.92%		
51–60	496	30.43%	250	30.19%		
≥60	625	38.34%	322	38.89%		
Sex					0.432	0.014
Female	361	22.15%	195	23.55%		
Male	1269	77.85%	633	76.45%		
Cancer location	1630		828		0.613	0.042
Buccal	715	43.87%	379	45.77%		
Gingival, hard palate, and oral tongue	804	49.33%	391	47.22%		
Lip	111	6.81%	58	7.00%		
Pathologic AJCC stage					0.960	0.001
I	479	29.39%	240	28.99%		
II	134	8.22%	68	8.21%		
III	421	25.83%	214	25.85%		
IV	596	36.56%	306	36.96%		
Income (NT$)	1630		828		0.082	0.129
Low income	17	1.04%	19	2.29%		
10,000–19,999	573	35.15%	286	34.54%		
≤20,000	411	25.21%	209	25.24%		
20,001–30,000	193	11.84%	81	9.78%		
30,001–45,000	83	5.09%	36	4.35%		
>45,000	353	21.66%	197	23.79%		
Urbanization					0.972	0.001
Rural	554	33.99%	282	34.06%		
Urban	1076	66.01%	546	65.94%		
CCI scores						
Mean (SD)	0.77 ± 1.05		0.93 ± 1.30		0.001	0.138
Median (IQR (Q1–Q3))	0.00 (0.00–1.00)		0.00 (0.00–2.00)		0.076	
CCI scores	1630		828		0.670	0.009
0	934	57.30%	467	56.40%		
≥1	696	42.70%	361	43.60%		
CCI comorbidities						
Congestive heart failure	65	3.99%	40	4.83%	0.329	0.008
Dementia	14	0.86%	9	1.09%	0.579	0.002
Chronic pulmonary disease	309	18.96%	161	19.44%	0.771	0.005
Rheumatic disease	16	0.98%	7	0.85%	0.740	0.001
Liver disease	307	18.83%	195	23.55%	0.006	0.047
Diabetes with complications	70	4.29%	31	3.74%	0.516	0.006
Hemiplegia and paraplegia	0	0.00%	1	0.12%	0.161	0.001
Renal disease	57	3.50%	39	4.71%	0.142	0.012
AIDS	1630	100.00%	828	100.00%		0.000
Alcohol liver diseases	311	19.08%	229	27.66%	<0.001	0.086
Cigarette smoking	537	32.94%	412	49.76%	<0.001	0.168
Betel nut chewing	326	20.00%	260	31.40%	<0.001	0.114
Adjuvant RT	541	33.19%	275	33.21%	0.482	0.001
Adjuvant CCRT	1089	66.81%	553	66.79%	0.481	0.001
Outcomes						
Mean (SD) follow-up	5.42 ± 4.63		4.58 ± 3.31		<0.001	
Median (IQR (Q1–Q3)) follow-up	5.14 (1.28–8.75)		4.48 (0.86–5.53)		<0.001	
All-cause death	724	44.42%	430	51.93%	<0.001	
Locoregional recurrence	387	23.74%	233	28.14%	<0.001	
Metastasis	253	15.52%	141	17.03%	<0.001	

Abbreviations: HR, hazard ratio; CI, confidence interval; AJCC, American Joint Committee on Cancer; NTD, New Taiwan Dollar; ref., reference group; CCI, Charlson comorbidity index; RT, radiotherapy; CCRT, concurrent chemoradiotherapy; SD, standard deviation; IQR, interquartile range; AIDS, acquired immunodeficiency syndrome.

**Table 2:** Univariable and multivariable Cox proportional regression of all-cause mortality between patients with oral cavity squamous cell carcinoma with and without sleep disorders.

	Crude HR (95%CI)		*p*-Value	Adjusted HR * (95%CI)		*p*-Value
Sleep disorder (ref. no.)						
Yes	1.32	(1.16–1.50)	0.001	1.19	(1.04–1.36)	0.011
Age group, years (ref. ≤50)						
51–60	1.22	(1.03–1.44)	0.018	1.22	(1.03–1.45)	0.020
>60	1.90	(1.64–2.20)	0.001	1.67	(1.42–1.98)	<0.001
Sex (ref. female)						
Male	1.70	(1.46–1.99)	0.001	1.76	(1.50–2.06)	<0.001
Income, NT$ (ref. low income)						
10,000–19,999	0.63	(0.40–1.00)	0.048	0.67	(0.42–1.08)	0.101
≤20,000	0.61	(0.39–0.95)	0.030	0.69	(0.44–1.10)	0.120
20,001–30,000	0.49	(0.31–0.77)	0.002	0.52	(0.33–0.83)	0.007
30,001–45,000	0.33	(0.20–0.54)	0.000	0.42	(0.25–0.70)	0.001
>45,000	0.24	(0.14–0.43)	0.000	0.28	(0.16–0.51)	0.000
Urbanization (ref. rural)						
Urban	0.84	(0.74–0.95)	0.007	0.92	(0.81–1.05)	0.198
Cancer location (ref. buccal)						
Gingival, hard palate, and tongue	0.93	(0.82–1.05)	0.218	1.06	(0.92–1.22)	0.440
Lip	0.77	(0.60–0.99)	0.041	0.91	(0.71–1.17)	0.471
AJCC stage (ref. stage I)						
II	1.47	(1.11–1.95)	0.0075	1.37	(1.03–1.82)	0.029
III	2.36	(1.96–2.85)	0.001	2.44	(2.02–2.95)	<0.001
IV	2.45	(2.05–2.93)	0.001	2.86	(2.36–3.46)	<0.001
CCI (ref. 0)						
≥1	1.45	(1.29–1.64)	0.001	1.19	(1.05–1.36)	0.007
Cigarette smoking (ref. no.)	1.68	(1.46–1.93)	0.001	1.35	(1.16–1.58)	<0.001
Betel nut chewing (ref. no.)	1.58	(1.39–1.79)	0.001	1.07	(1.02–1.14)	0.008
Alcohol liver diseases (ref. no.)	1.69	(1.48–1.94)	0.001	1.27	(1.09–1.48)	0.002
Adjuvant RT	0.85	(0.49–0.91)	0.001	0.97	(0.95–1.20)	0.296
Adjuvant CCRT	0.81	(0.55–0.95)	0.001	0.92	(0.80–1.05)	0.228

Abbreviations: HR, hazard ratio; CI, confidence interval; AJCC, American Joint Committee on Cancer; NTD, New Taiwan Dollars; ref., reference group; CCI, Charlson comorbidity index; RT, radiotherapy; CCRT, concurrent chemoradiotherapy. * All covariates mentioned in Table 2 were adjusted.

**Table 3:** Univariable and multivariable Cox proportional regression of locoregional recurrence between patients with oral cavity squamous cell carcinoma with and without sleep disorders.

	Crude HR (95%CI)		*p*-Value	Adjusted HR * (95%CI)		*p*-Value
Sleep disorder (ref. no.)						
Yes	1.41	(1.19–1.67)	0.001	1.47	(1.23–1.75)	<0.001
Age group, years (ref. ≤50)						
51–60	1.14	(0.94–1.38)	0.196	1.11	(0.91–1.36)	0.310
>60	0.91	(0.74–1.10)	0.327	1.00	(0.80–1.25)	0.993
Sex (ref. female)						
Male	2.74	(2.14–3.50)	0.001	1.84	(1.43–2.37)	<0.001
Income, NT$ (ref. low income)						
10,000–19,999	1.09	(0.45–2.68)	0.846	1.09	(0.44–2.70)	0.861
≤20,000	1.29	(0.53–3.12)	0.580	1.16	(0.47–2.84)	0.748
20,001–30,000	1.59	(0.65–3.87)	0.306	1.42	(0.58–3.48)	0.447
30,001–45,000	1.44	(0.58–3.55)	0.434	1.40	(0.56–3.49)	0.475
>45,000	0.87	(0.33–2.29)	0.779	0.97	(0.36–2.59)	0.953
Urbanization (ref. rural)						
Urban	0.85	(0.72–1.00)	0.055	1.05	(0.88–1.25)	0.607
Cancer location (ref. buccal)						
Gingival, hard palate, and tongue	1.15	(0.54–1.82)	0.329	1.11	(0.91–1.22)	0.452
Lip	1.08	(0.69–1.68)	0.738	1.03	(0.66–1.61)	0.901
AJCC stage (ref. stage I)						
II	1.25	(1.00–1.56)	0.055	1.35	(1.08–1.70)	0.010
III	1.66	(1.27–2.16)	0.0002	1.64	(1.25–2.15)	<0.001
IV	2.04	(1.69–2.47)	0.001	1.93	(1.60–2.33)	<0.001
CCI (ref. 0)						
≥1	1.09	(0.92–1.28)	0.3275	1.15	(0.97–1.37)	0.111
Cigarette smoking (ref. no.)	1.21	(1.02–1.43)	0.0251	1.25	(1.02–1.54)	0.030
Betel nut chewing (ref. no.)	1.29	(1.14–1.47)	0.001	1.26	(1.08–1.48)	0.003
Alcohol liver diseases (ref. no.)	1.39	(1.14–1.69)	0.0009	1.04	(0.83–1.30)	0.737
Adjuvant RT	0.44	(0.22–0.89)	0.022	0.69	(0.49–0.79)	0.009
Adjuvant CCRT	0.30	(0.13–0.73)	0.008	0.56	(0.41–0.83)	0.003

Abbreviations: HR, hazard ratio; CI, confidence interval; AJCC, American Joint Committee on Cancer; NTD, New Taiwan Dollar; ref., reference group; CCI, Charlson comorbidity index; RT, radiotherapy; CCRT, concurrent chemoradiotherapy. * All covariates mentioned in Table 2 were adjusted.

**Table 4:** Univariable and multivariable Cox proportional regression of distant metastasis between patients with oral cavity squamous cell carcinoma with and without sleep disorders.

	Crude HR (95%CI)		*p*-Value	Adjusted HR * (95%CI)		*p*-Value
Sleep disorder (ref. no.)						
Yes	1.29	(1.03–1.60)	0.025	1.15	(1.02–1.44)	0.025
Age group, years (ref. ≤50)						
51–60	1.02	(0.79–1.31)	0.873	1.04	(0.8–1.34)	0.795
>60	1.02	(0.8–1.30)	0.895	1.18	(0.9–1.56)	0.236
Sex (ref. female)						
Male	2.02	(1.53–2.67)	0.001	1.63	(1.22–2.18)	0.001
Income, NT$ (ref. low income)						
10,000–19,999	3.09	(0.43–22.25)	0.263	4.45	(0.61–32.46)	0.141
≤20,000	3.76	(0.53–26.89)	0.187	4.75	(0.66–34.37)	0.123
20,001–30,000	4.22	(0.59–30.24)	0.152	5.01	(0.69–36.25)	0.111
30,001–45,000	2.68	(0.37–19.55)	0.331	3.39	(0.46–25.12)	0.231
>45,000	2.33	(0.31–17.62)	0.414	2.91	(0.38–22.35)	0.305
Urbanization (ref. rural)						
Urban	0.84	(0.68–1.04)	0.101	0.88	(0.71–1.10)	0.258
Cancer location (ref. buccal)						
Gingival, hard palate, and tongue	1.09	(0.88–1.34)	0.442	1.35	(0.95–1.74)	0.219
Lip	0.74	(0.47–1.15)	0.184	0.94	(0.66–1.63)	0.877
AJCC stage (ref. stage I)						
II	2.00	(1.00–4.02)	0.051	1.89	(1.14–3.80)	0.0076
III	3.12	(1.49–4.26)	0.001	3.31	(1.6–4.62)	<0.001
IV	7.00	(5.15–9.42)	0.001	8.44	(6.58–9.55)	<0.001
CCI (ref. 0)						
≥1	1.25	(0.92–1.54)	0.338	1.21	(0.98–1.51)	0.182
Cigarette smoking (ref. no.)	1.16	(0.98–1.37)	0.079	1.12	(0.94–1.34)	0.210
Betel nut chewing (ref. no.)	1.17	(0.95–1.45)	0.141	1.05	(0.83–1.34)	0.667
Alcohol liver diseases (ref. no.)	1.13	(0.95–1.34)	0.164	1.02	(0.83–1.25)	0.843
Adjuvant RT	0.85	(0.4–1.80)	0.679	1.41	(0.65–3.04)	0.388
Adjuvant CCRT	0.86	(0.43–1.72)	0.661	1.49	(0.73–3.05)	0.275

Abbreviations: HR, hazard ratio; CI, confidence interval; AJCC, American Joint Committee on Cancer; NTD, New Taiwan Dollar; ref., reference group; CCI, Charlson comorbidity index; RT, radiotherapy; CCRT, concurrent chemoradiotherapy. * All covariates mentioned in Table 2 were adjusted.

## Data Availability

The datasets supporting the study conclusions are included in this manuscript and its supplementary files.

## Supplementary Materials

The following supporting information can be downloaded at: https://www.mdpi.com/article/10.3390/cancers14143420/s1, Figure S1: Kaplan–Meier cumulative locoregional recurrence curves of propensity score–matched patients with oral cavity squamous cell carcinoma with and without sleep disorders; Figure S2: Kaplan–Meier cumulative distant metastasis curves of propensity score–matched patients with oral cavity squamous cell carcinoma with and without sleep disorders.
