# Supplementary material for: Association between Preexisting Sleep Disorders and Oncologic Outcome in Patients with Oral Cavity Squamous Cell Carcinoma: A Nationwide Propensity Score—Matched Population-Based Cohort Study
Source: Cancers (Basel). 2022 Jul 14;14(14):3420. doi: 10.3390/cancers14143420 (PMC9318372; doi:10.3390/cancers14143420)
Supplement: Supplementary file 1 [file cancers-14-03420-s001.zip › cancers-1792573-supplementary.pdf]

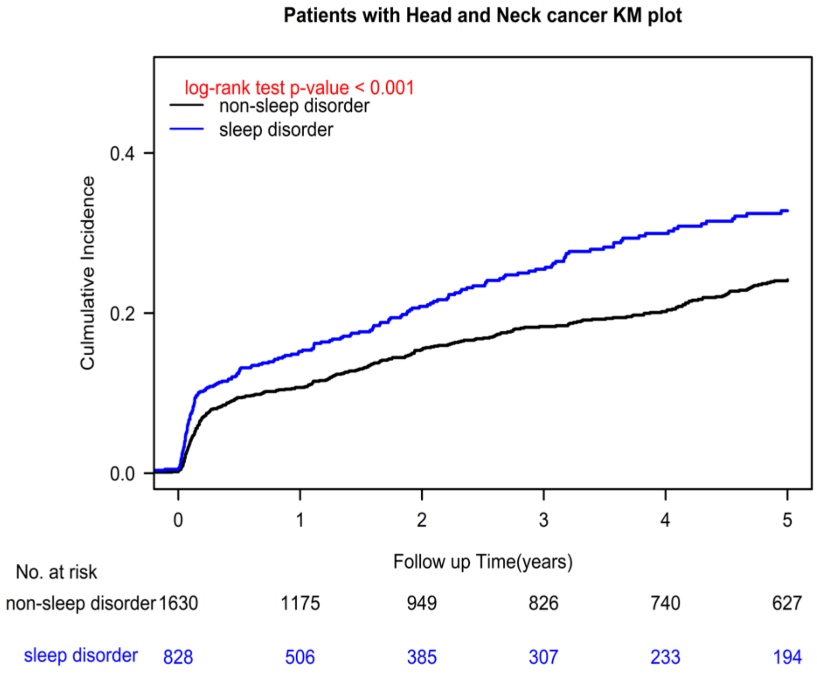

Supplemental Figure S1. Kaplan–Meier cumulative locoregional recurrence curves of propensity score–matched patients with oral cavity squamous cell carcinoma with and without sleep disorders.

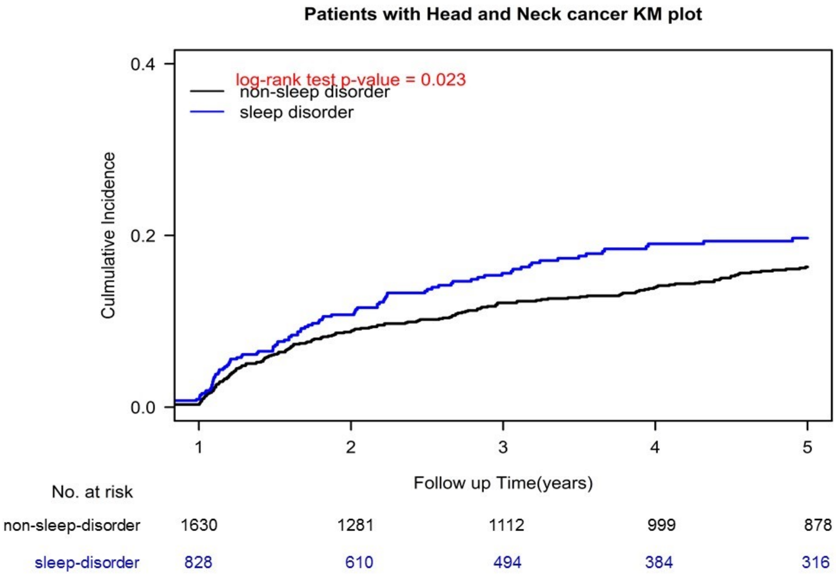

Supplemental Figure S2. Kaplan–Meier cumulative distant metastasis curves of propensity score–matched patients with oral cavity squamous cell carcinoma with and without sleep disorders.
